# Baicalein reduces angiogenesis in the inflammatory microenvironment via inhibiting the expression of AP-1

**DOI:** 10.18632/oncotarget.13669

**Published:** 2016-11-26

**Authors:** Yujie Huang, Zhaorui Miao, Yang Hu, Yang Yuan, Yuxin Zhou, Libin Wei, Kai Zhao, Qinglong Guo, Na Lu

**Affiliations:** ^1^ State Key Laboratory of Natural Medicines, Jiangsu Key Laboratory of Carcinogenesis and Intervention, Jiangsu Key Laboratory of Drug Design and Optimization, China Pharmaceutical University, Nanjing 210009, People's Republic of China

**Keywords:** baicalein, angiogenesis, inflammatory, AP-1

## Abstract

Increasing clinical and experimental studies have demonstrated that refractory chronic inflammation will result in malignant tumor and anti-angiogenic therapy may be an effective way to thwart the progression. Baicalein, one of the major active flavanoids found in *Scutellaria baicalensis* Georgi, has been exhibited potent anti-inflammation and anti-tumor effects by reducing angiogenesis. However, the exact mechanism of baicalein on endothelial cells in inflammatory microenvironment was not clear yet. Here, we investigated the anti-angiogenic effect of baicalein by incubating human umbilical vein endothelial cells (HUVECs) with THP-1 conditioned medium *in vitro*. The tube formation of HUVECs and microvessel outgrowth of rat aorta were attenuated, as well as the number of newly formed blood vessels in chicken chorioallantoic membrane (CAM) was reduced by baicalein. This anti-angiogenic effect was mainly on account of the inhibited motility, migration and invasion of HUVECs. In addition, mechanistic studies showed that baicalein could bind to AP-1 directly and the expression of c-Jun and c-Fos in HUVECs was reduced, accompanied by their increased proteasomal degradation. Besides, baicalein suppressed the nuclear translation, heterodimer formation and DNA binding affinity of c-Jun and c-Fos. What's more, the anti-angiogenic effect of baicalein was further confirmed by matrigel plug assay *in vivo*. Taken together, our study demonstrated that baicalein could exert its anti-angiogenic effect in the inflammation microenvironment via inhibiting the transcriptional activity of AP-1, which suggested that baicalein might be an alternative treatment against refractory chronic inflammation.

## INTRODUCTION

Angiogenesis is defined as the formation and growth of new blood vessels from the pre-existing vasculature, which physiologically takes place during embryogenesis and wound healing, as well as in the female reproductive cycle [1]. However, the dysregulated formation of new blood vessels contributes to ischemic, eye diseases, especially solid tumor growth and inflammation [2]. The chronic inflammation causes substantial tissue damage, which might create pro-carcinogenesis conditions followed by the rapid development of cancer [3]. In this situation, pathological angiogenesis promotes the recruitment of inflammatory cells continuously, thereby exacerbating inflammation and damage, for instance, the formation of pannus is one of the pathological hallmarks of rheumatoid arthritis [4], an inflammatory connective tissue mass is rich in blood vessels and apparently dependent on angiogenic factors [5], the development of malignancy is promoted by angiogenesis in inflammatory conditions [6, 7]. In return, inflammatory cells, including neutrophils, mast cells, and macrophages, are involved in promoting angiogenesis by secreting a plethora of proangiogenic factors, such as vascular endothelial growth factor (VEGF), tumor necrosis factor-α (TNF-α), and other cytokines [8]. To date, there are more than 500 articles showing the close relationship between angiogenesis with inflammatory microenvironment and therapeutic induction of angiogenesis has been tested, which would be a promising choice to suppress angiogenesis in the treatment of inflammatory diseases and cancer [9, 10]. Therefore, extensive research is warranted to discover effective agents which can inhibit angiogenesis induced by inflammatory stimulus.

The activator protein-1 (AP-1) transcription factor has represented a paradigm for gene regulation implicated in cancer and inflammatory diseases [11] which is closely associated with angiogenesis by regulating the expression of pro-anigogenic factors including VEGF, matrix metalloproteinases (MMPs) and inflammatory cytokines [12, 13]. AP-1 consists of diverse hetero or homo-dimeric complex comprising proteins from Fos (c-Fos, Fos B, Fra-1 and Fra-2), Jun (c-Jun, Jun B and Jun D), ATF (ATF2, LRF1/ATF3, B-ATF, JDP1 and JDP2) and Maf (c-Maf, Maf B, Maf A, Maf G/F/K and Nrl) subfamilies, among which Jun and Fos subfamilies are the major AP-1 proteins [14–15]. The dimers are translocated into the nucleus to bind DNA and initiates downstream target genes transcription related to cell-proliferation, transformation, cancer and inflammation [16].

The matrix metalloproteinases (MMPs) are the principal enzymes in extracellular matrix (ECM) degradation, and the degradation of blood vessel basement membrane by MMPs is believed to be the beginning of angiogenesis [17]. Subsequently, MMPs catalytically trigger the migration of endothelial cells (ECs) and play an essential role in the development of neovasculature during angiogenesis [18]. According to the substrate-based classification, MMPs can be divided into collagenases, stromelysins, elastases and gelatinases. Among all of the MMPs, MMP-2 (gelatinase A) and MMP-9 (gelatinase B) are considered to play a leading role in cleaving the ECM [19]. In addition, MMP-2 and MMP-9, predominately expressed in the ECs, are directly involved in the migration of ECs and vascular remodeling during angiogenesis [20].

Baicalein (5, 6, 7-trihydroxy-2-phenyl-4H-1-benzopyran-4-one) is one of the major active flavanoids found in traditional Chinese medicine *Scutellaria baicalensis* Georgi, an herb widely used to treat ischemia and various inflammatory diseases [21]. It has been reported that baicalein suppresses VEGF and basic fibroblast growth factor (bFGF)-induced angiogenesis via inhibiting the proliferation of ECs [22, 23]. Except for proliferation, EC activation is also one of the necessary steps during neovascularization, as well as in helping vessel normalization and function. To explore the anti-angiogenic effect and its exact mechanism more comprehensively, the effect of baicalein on EC activation needs to be studied. Besides, previous studies have showed that baicalein exhibits potent anti-inflammatory by inhibiting 12/15-lipoxygenase (12/15-LOX) [24] and the anti-inflammation treatments of baicalein are relying on the decreased secretion of inflammatory factors, such as reduction of IL-1 and TNF-α [25, 26]. As anti-angiogenic therapy may be an effective way to thwart the refractory chronic inflammation, whether baicalein could affect angiogenesis in the inflammatory microenvironment, along with the molecular mechanisms, warrants further investigations. Therefore, the inflammation-induced angiogenesis models were established, in which ECs were incubated by the conditioned media of LPS-stimulated THP-1 cells instead of only one stimulator, such as VEGF and bFGF, to reasonably simulate the *in vivo* microenvironment.

In our study, we investigated the anti-angiogenic effect of baicalein in inflammatory microenvironment and the potential mechanisms. We incubated human umbilical vein endothelial cells (HUVECs) with THP-1 conditioned medium (THP-1 CM) to evaluate the inhibition effect of baicalein on angiogenesis *in vitro*, which were confirmed by matrigel plug assay *in vivo.* Further mechanism study revealed that baicalein inhibited angiogenesis by inhibiting the expression, nuclear translocation and DNA binding affinity of AP-1 in THP-1 CM-induced HUVECs. Taken together, these results suggested that baicalein inhibited angiogenesis in inflammatory microenvironment through a potential mechanism attributed to inhibiting AP-1 signaling and baicalein may serve as a candidate in the treatment of refractory chronic inflammation.

## RESULTS

### Baicalein inhibited THP-1 CM-induced angiogenesis *in vitro* and *ex vivo*

**Figure 1:**
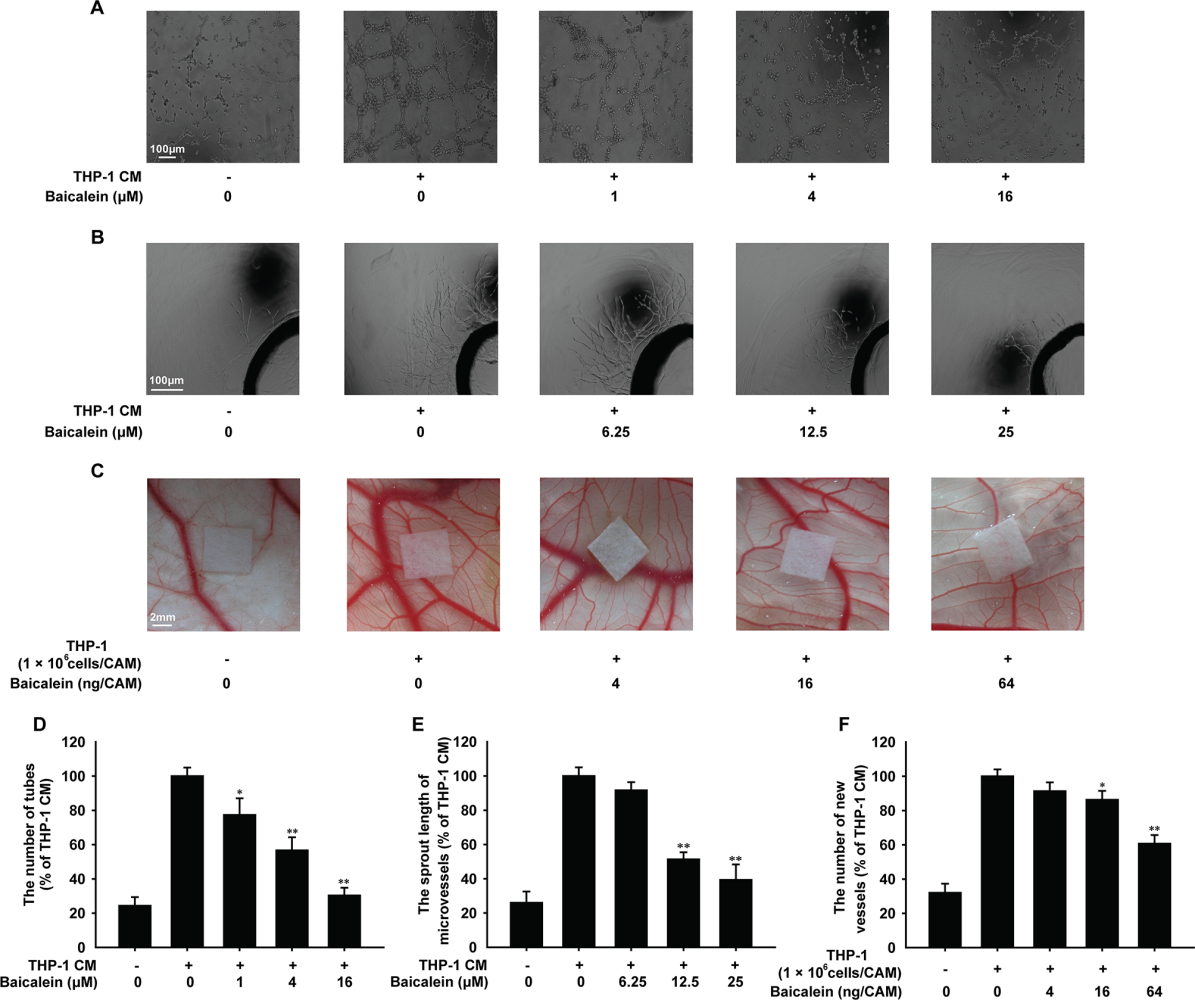
Effects of baicalein on THP-1 CM-induced angiogenesis *in vitro* and *ex vivo* (**A**) The tube formation of HUVECs induced by THP-1 CM. HUVECs were pretreated with regular medium containing 50% THP-1CM with various concentrations of baicalein (0, 1, 4 and 16 μM) for 24 h or regular medium in control group. (**B**) Microvessel sprouting of rat aortic ring stimulated by THP-1 CM. After 3 days of microvessel-like structures growth of rat aortic ring, the growth media containing 50% THP-1 CM with baicalein was added into each group as indicated. (**C**) Effect of baicalein on the angiogenesis *ex vivo* was verified in CAM model. The LPS-activated THP-1 cells (1 × 10^6^ cells/embryo) were placed on the exposed CAM and the sterilized filter paper disks (5 mm × 5 mm) saturated with various concentrations of baicalein were added as indicated. (**D**) Tubular structures were quantified by manual counting the tube numbers, and five randomly chosen fields were analyzed for each well. (**E**) Quantification of the microvessel growth of rat aortic rings. (**F**) The CAM assay was quantified by counting the number of new grown vessels on digitalized pictures. Each experiment was performed at least three times. Data are presented as mean ± SD. The comparisons were made relative to THP-1 CM-treated group and significance of difference is indicated as *P < 0.05, **P < 0.01.

To evaluate the effect of baicalein on angiogenesis *in vitro*, we firstly performed the tube formation assay. As shown in Figure 1A, upon exposure to THP-1 CM, the formation of elongated and robust tube-like structures by HUVECs increased significantly. Compared with the THP-1 CM-stimulated group, baicalein at 1, 4 and 16 μM resulted in the inhibition of tube formation by 22.7%, 43.3%, and 69.7%, respectively. Then, the rat aortic ring assay was adopted, which could mimic several stages in angiogenesis, including endothelial cell proliferation, migration and tube formation. THP-1 CM promoted the formation of microvessel outgrowth from explants of rat aorta obviously, which was shown in Figure 1B. However, the treatment of baicalein (1, 4, 16 μM) inhibited the growth of microvessel, and compared with THP-1 CM stimulated group the inhibition percentage was 8.3%, 48.7% and 60.7%, respectively. CAM assay was used as a unique *ex vivo* model to further investigate the anti-angiogenic effect of baicalein on the process of new blood vessel formation. Compared with control group, there were more new blood vessels formed in THP-1 cells-induced group (Figure 1C). Whereas, the quantitative analysis indicated that when baicalein (4, 6, 64 ng/CAM) was added, the number of newly formed blood vessels decreased and the inhibition efficiency was 8.7%, 13.7%, and 39.3%, respectively. All these results indicated that baicalein inhibited angiogenesis induced by THP-1 CM *in vitro* and *ex vivo*.

### Baicalein inhibited THP-1 CM-induced migration and invasion of HUVECs

**Figure 2:**
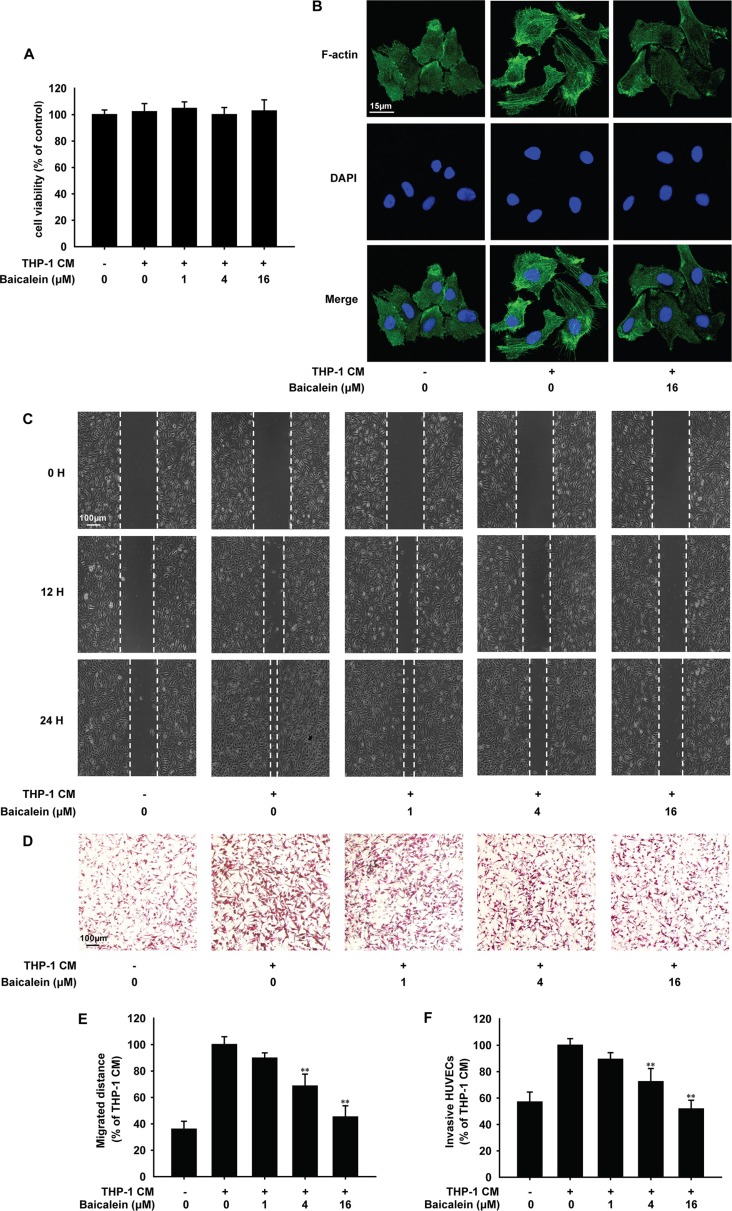
Effects of baicalein on THP-1 CM induced cytoskeleton remodeling and migration of HUVECs HUVECs were incubated with regular medium in control group or regular medium containing 50% THP-1CM with various concentrations of baicalein (0, 1, 4 and 16 μM) for 24 h. (**A**) The viability of HUVECs was examined by MTT assay. (**B**) Cytoskeleton remodeling of HUVECs was tested by immunofluorescence. (**C**) Migration of HUVECs was tested by wound healing assay. (**D**) Effect of baicalein on THP-1 CM-induced HUVEC invasion was tested by endothelial cell invasion assay. (**E**) Wound healing was quantified by measuring the migrated distance of HUVECs. (**F**) Migrated cells were quantified by manual counting and five randomly chosen fields were analyzed for each group. Each experiment was performed at least three times. Data are presented as mean ± SD. The comparisons were made relative to THP-1 CM-treated group and significance of difference is indicated as *P < 0.05, **P < 0.01.

Angiogenesis is often characterized by excessive proliferation, increased motility and migration of vascular endothelial cells. The MTT assay (Figure 2A) showed that treatment with baicalein for 24 h had no effect on the proliferation of HUVECs, which indicated the suppressive effect of baicalein on THP-1 CM-induced angiogenesis was not due to the decreased vitality of endothelial cells.

Then we investigated the effect of baicalein on HUVECs migration and invasion. Actin filaments were stained by FITC-phalloidin (Figure 2B). F-actin localized mainly at the cell periphery under normal condition, which became less prominent and the cytoplasmic stress fibers formed with the stimulation of THP-1 CM. When treated with 16 μM baicalein, cytoplasmic stress fibers were blocked. Wound-healing assay (Figure 2C) indicated that THP-1 CM stimulated apparent migration of HUVEC monolayers after 24 h. With the treatment of baicalein (1, 4, 16 μM), HUVECs migrated less and slowly, and the inhibition rate was 10.2%, 31.3% and 54.8%, respectively. To investigate whether baicalein inhibits HUVEC invasion, the matrigel invasion assay was performed. As shown in Figure 2D, the number of invasive HUVECs was decreased by baicalein in a concentration-dependent manner and compared with THP-1 CM-treated group, the inhibition rate of baicalein at 1, 4, 16 μΜ was 10.4%, 28.2% and 61.3%, respectively. These results showed that baicalein suppressed THP-1 CM-induced migration and invasion of HUVECs.

**Figure 3:**
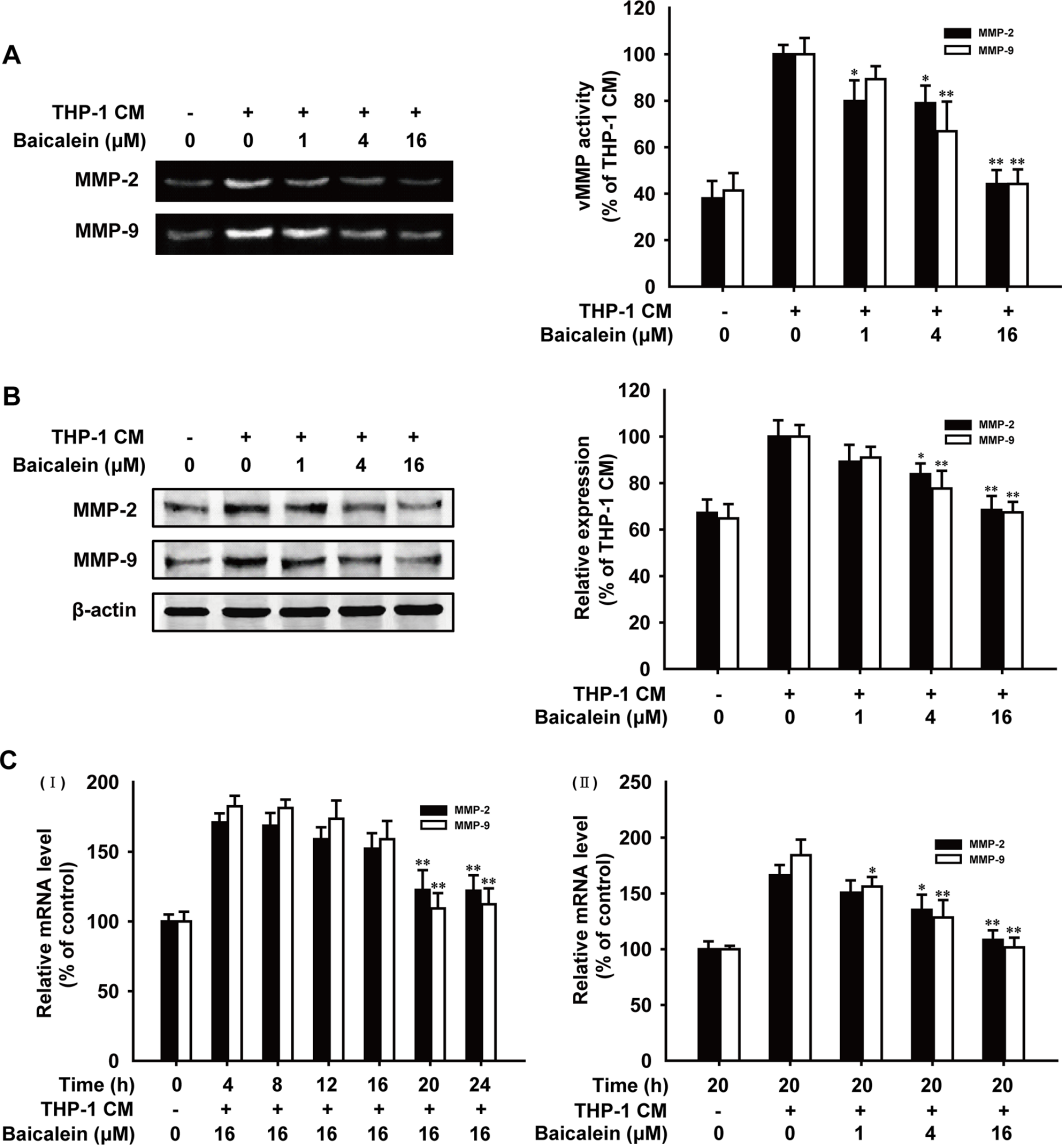
Effects of baicalein on the activity and expression of MMP-2 and MMP-9 in THP-1 CM-stimulated HUVECs (**A**) The conditioned medium of HUVECs pretreated with different concentrations of baicalein as indicated was collected and then analyzed for the enzyme activity of MMP-2 and MMP-9 by gelatin zymography. (**B**) Total protein expression of MMP-2 and MMP-9 in HUVECs were detected by western blot analysis using specific antibodies and β-actin was used as loading control. (**C**) mRNA level of MMP-2 and MMP-9 were measured by real-time PCR after HUVECs were incubated with indicated times (I) and concentrations of baicalein (II). Each experiment was performed at least three times. Data are presented as mean ± SD. The comparisons were made relative to THP-1 CM-treated group and significance of difference is indicated as *P < 0.05, **P < 0.01.

Gelatin zymography was carried out to investigate the effect of baicalein on gelatinolytic activity of MMP-2 and MMP-9 secreted by HUVECs. As shown in Figure 3A, quantification analysis of the gelatinolytic activity date indicated that when cells were treated with baicalein (1, 4, 16 μM), MMP-2 activity reduced by 20.1%, 21.1%, 55.8% and MMP-9 activity reduced by 10.6%, 33.1%, 55.8%, respectively. Western blot analysis (Figure 3B) showed that the expression of MMP-2 and MMP-9 in baicalein treated groups decreased in a concentration-dependent manner, and the inhibition percentage of baicalein (16 μM) treatment was 31.6% and 32.6%, respectively. The mRNA levels of MMP-2 and MMP-9 were further elucidated by real-time PCR. As shown in Figure 3C, the inhibition effect of baicalein on MMP-2/9 gene expression was observed in time and concentration-dependent manners. The inhibition rate of MMP-2 and MMP-9 was 58.0% and 82.6%, respectively, with the treatment of baicalein (16 μM) for 20 h. All of these results demonstrated that baicalein suppressed THP-1 CM-induced activity and expression of MMP-2 and MMP-9 in HUVECs.

### Baicalein suppressed the expression of AP-1 in THP-1 CM-stimulated HUVECs

**Figure 4:**
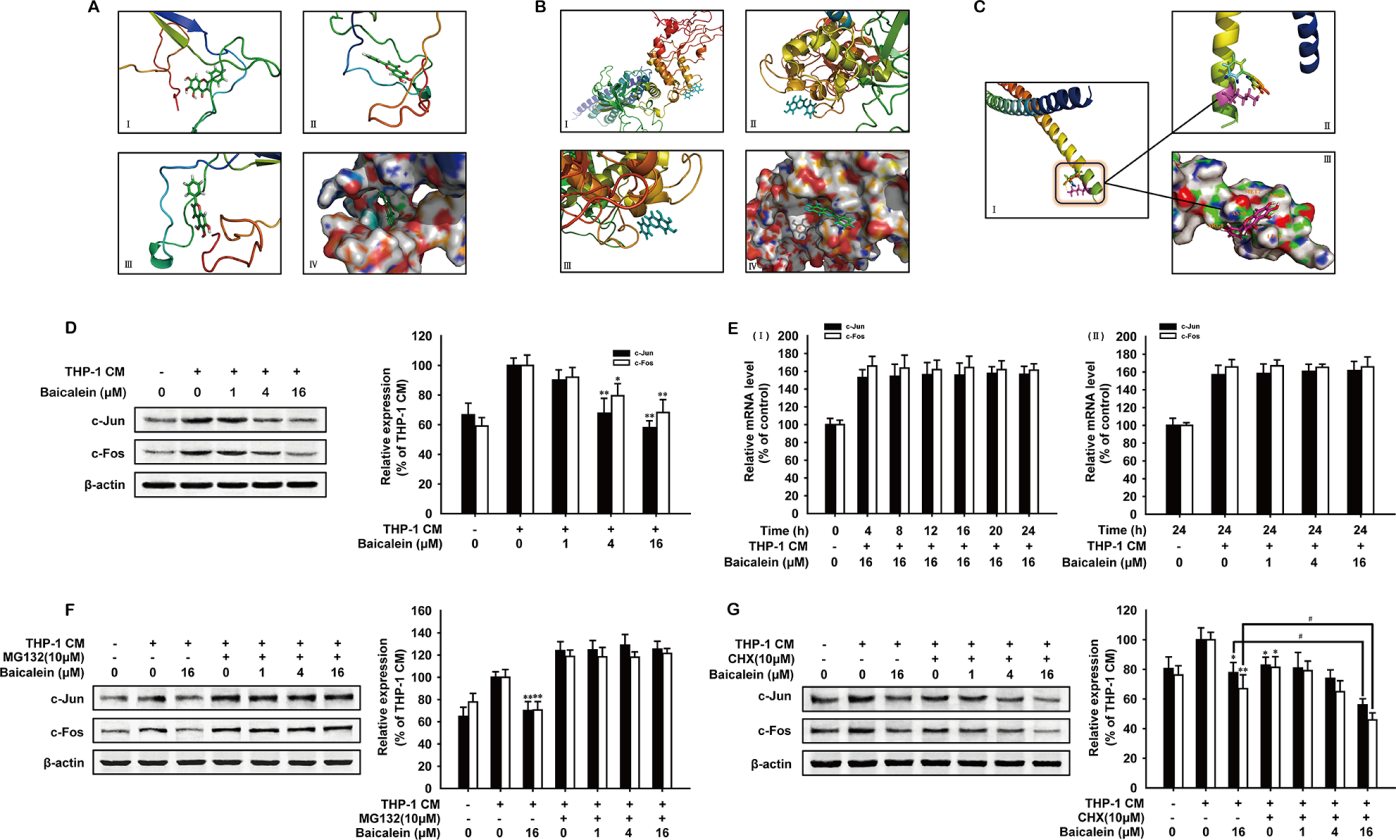
Effects of baicalein on the expression of AP-1 in THP-1 CM-stimulated HUVECs The binding mode picture of baicalein with NF-κB (**A**), STAT-3 (**B**) and AP-1 (**C**) were shown according to the docking study. (C-I) shows the binding site of baicalein with AP-1 and the hydrogen bond with Lys5 site was marked in the magnified picture (C-II). (**D**) Total protein expression of c-Jun and c-Fos in HUVECs were detected by western blot analysis using specific antibodies. β-actin was used as an internal control. HUVECs were exposed to regular medium alone or regular medium containing 50% THP-1 CM with baicalein at indicated concentrations for 24 h. (**E**) The mRNA level of c-Jun and c-Fos were measured by real-time PCR after HUVECs were incubated with indicated times (I) and concentrations of baicalein (II). (**F**–**G**) The expression of c-Jun and c-Fos were detected by Western blot analysis. HUVECs were incubated with various concentrations of baicalein and MG-132 (10 μM) (F) or CHX (10 μM) (G) for 24 h. Each experiment was performed at least three times. Data are presented as mean ± SD. The comparisons were made relative to THP-1 CM-treated group and significance of difference is indicated as *P < 0.05, **P < 0.01.

NF-κB, STAT-3 and AP-1 are three main transcription factors activated frequently in inflammation microenvironment, then increase the expression of MMPs and promote angiogenesis [27, 28] To elucidate the anti-angiogenic mechanism of baicalein, we firstly applied bioinformatics to explore the direct interaction of baicalein with transcription factors by molecular modeling and docking studies. As shown in Figure 4A, the energy minimum molecular pose of baicalein had no molecular hydrogen bond with NF-κB and the molecular distance between ligand and the receptor was close enough to form a repulsive force. Baicalein was almost distant free from the tertiary structure of STAT-3 and neither could form a stable hydrogen bond (Figure 4B). However, the result in Figure 4C indicated that baicalein formed a stable hydrogen bond with AP-1 at the Lys5 site (Figure 4C-II). The aromatic ring branched chain of baicalein stretched into the hydrophobic pocket consisted of Lys5, Arg6, Met7, Arg8 and Asn9 (Figure 4C-III). Then we focused our attention to AP-1 and examined the effect of baicalein on the expression of c-Jun and c-Fos in HUVECs. As shown in Figure 4D, baicalein remarkably down-regulated the expression of c-Jun and c-Fos, while the mRNA level of c-Jun and c-Fos was not changed by baicalein (Figure 4E). In addition, the reduction effect of baicalein on c-Jun and c-Fos was abrogated with the addition of MG-132, which can block proteasome-mediated protein degradation (Figure 4F). CHX (cycloheximide) was used for inhibiting de novo protein synthesis. As shown in Figure 4G, baicalein decreased protein level of c-Jun and c-Fos more noticeably when the new c-Jun and c-Fos synthesis was inhibited by CHX. Compared with control group, the inhibition rate of c-Jun and c-Fos by baicalein (16 μM) was 22.2% and 33.0%, respectively, while in the presence of 10 mM CHX, the inhibition rate increased to 44.0% and 54.1% for groups treated with the same concentration of baicalein. These data suggested that baicalein could bind to AP-1 directly and suppress AP-1 expression.

### Baicalein decreased the nuclear accumulation and DNA binding affinity of AP-1 in THP-1 CM-induced HUVECs

**Figure 5:**
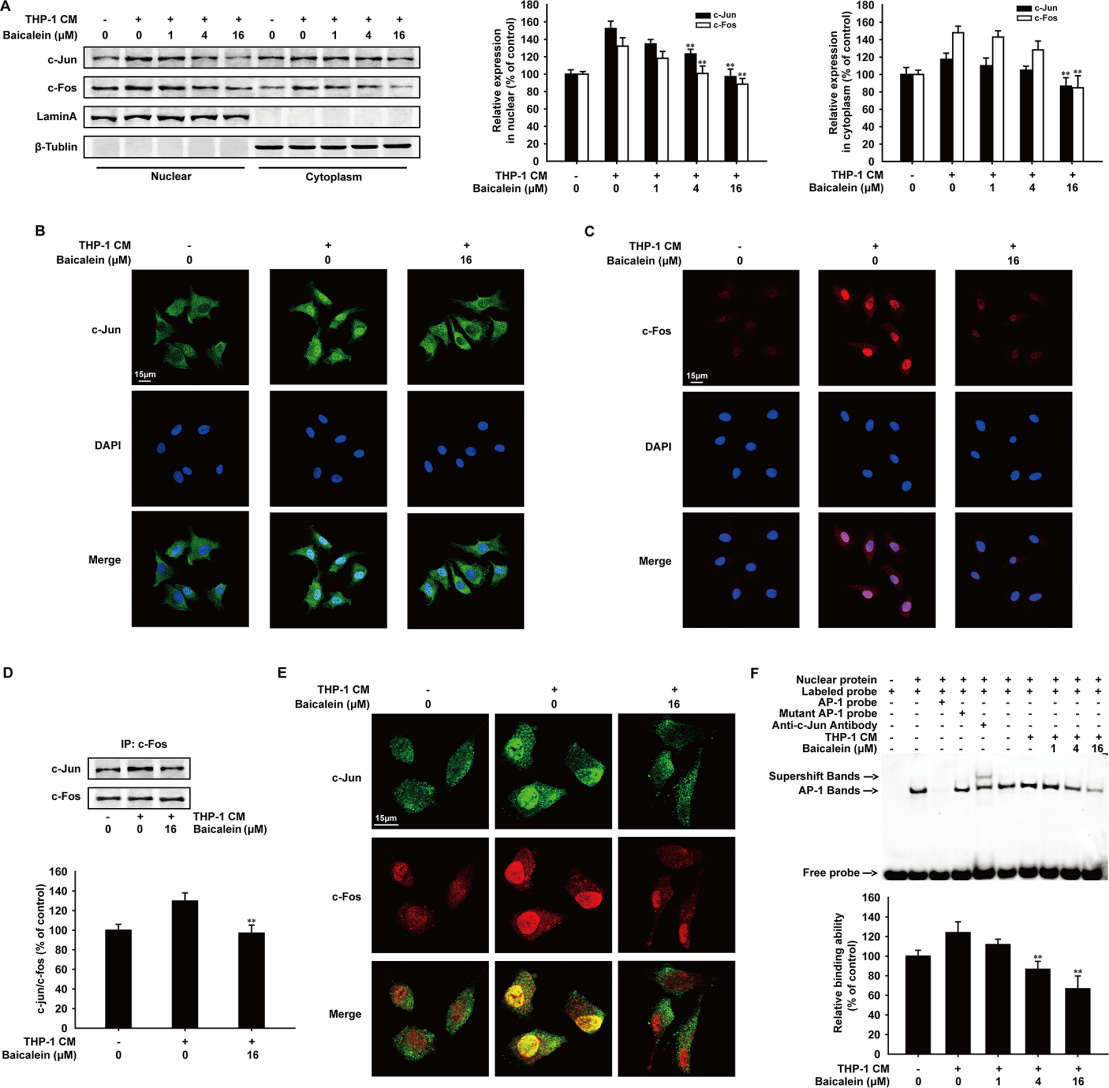
Effects of baicalein on THP-1 CM-induced nuclear tanslocation, dimer formation and binding activity with exogenous consensus DNA oligonucleotide of AP-1 (**A**) Western blot analysis of c-Jun and c-Fos expression in cytosolic and nuclear lysates of HUVECs incubated with baicalein (0, 1, 4, 16 μM) for 24 h. (**B**–**C**) The nuclear translocation of c-Jun (B) and c-Fos (C) was analyzed by Immunofluorescence confocal microscopy. (**D**) Equal amounts of cell lysates treated with/without baicalein (16 μM) were immunoprecipitated with anti-c-Fos antibody, followed by Western blot analysis with anti-c-Jun antibody. (**E**) Immunofluorescence was performed to analyze the heterodimer formation of c-Jun and c-Fos in HUVECs. (**F**) Nuclear extracts were prepared and subjected to EMSA to detect DNA-binding activity of AP-1. Each experiment was performed at least three times. Data are presented as mean ± SD. The comparisons were made relative to THP-1 CM-treated group and significance of difference is indicated as *P < 0.05, **P < 0.01.

The cytoplasmic accumulation, intracellular localization and dimer formation of AP-1 are essential for activating the transcription of target gens, including MMP-2 and MMP-9 [29]. The c-Jun and c-Fos protein expression in cytosolic and nuclear lysates of HUVECs was examined by Western blot analysis (Figure 5A). As the concentration of baicalein increased, c-Jun and c-Fos in cell nucleus were both decreased. Immunofluorescence (Figure 5B and 5C) also showed that the nuclear translocation of c-Jun and c-Fos were inhibited obviously by baicalein (16 μΜ). In addition, immunoprecipitation assay (Figure 5D) revealed that the combination of c-Jun and c-Fos induced by THP-1 CM was reduced by 32.8%. Meanwhile, the inhibition effect of baicalein on the heterodimer formation of c-Jun and c-Fos in cell nucleus was also confirmed by immunofluorescence (Figure 5E). To further explore the regulation mechanism between AP-1 and baicalein, we then investigated the DNA binding affinity of AP-1 in THP-1 CM-induced HUVECs by EMSA analysis (Figure 5F). The result showed that baicalein inhibited the binding activity of AP-1, and compared with THP-1 CM group, the inhibition percentage by baicalein (1, 4, 16 μM) was 12.1%, 37.4% and 57.3%, respectively. Collectively, we concluded that baicalein inhibited the nuclear translocation, dimer formation and DNA binding affinity of AP-1 in THP-1 CM-induced HUVECs.

### Baicalein inhibited angiogenesis via suppressing AP-1 signaling in HUVECs

**Figure 6:**
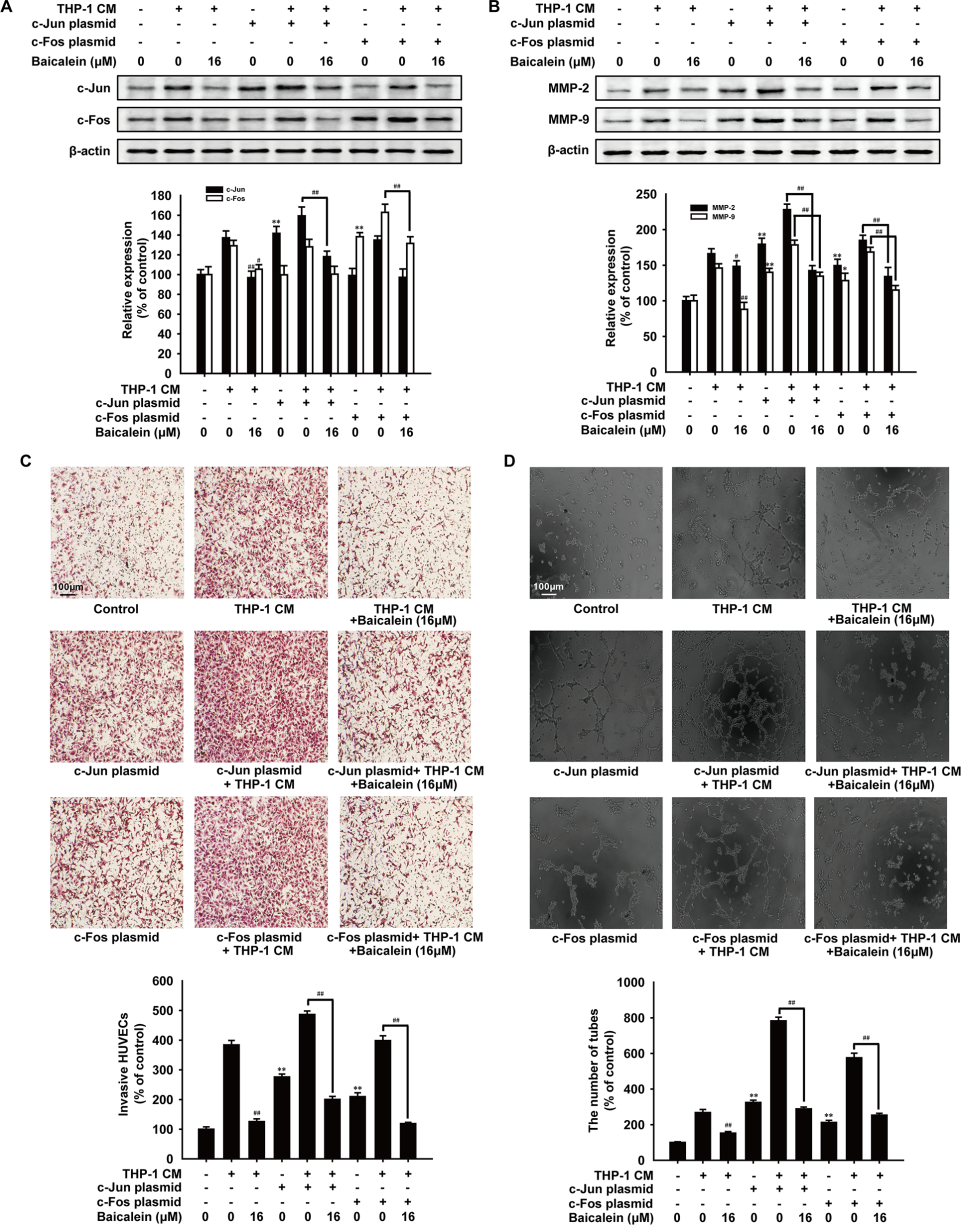
Effects of baicalein on angiogenesis after HUVECs were transfected with AP-1 plasmid HUVECs were transfected with c-Jun or c-Fos plasmid before treated with THP-1 CM with baicalein (16 μM) as indicated. (**A**) Protein levels of c-Jun and c-Fos were detected by Western blot using specific antibodies. (**B**) Protein levels of MMP-2 and MMP-9 were detected by Western blot using specific antibodies. β-actin was used as an internal control. (**C**) Invasion of HUVECs was tested by endothelial cell invasion assay. (**D**) Effect of baicalein on THP-1 CM-induced angiogenesis was tested by tube formation assay. Each experiment was performed at least three times. Data are presented as mean ± SD. *P < 0.05, **P < 0.01 compared with control group; ^#^*P* < 0.05, ^##^*P* < 0.01 compared with THP-1 CM-treated group.

To assess the role of AP-1 in the effect of baicalein on THP-1 CM-induced angiogenesis, HUVECs were transfected with c-Jun or c-Fos expression plasmid before treated with baicalein. As shown in Figure 6A and 6B, after cells were transfected with c-Jun or c-Fos expression plasmid, the level of c-Jun or c-Fos and the expression of MMP-2 and MMP-9 were both up-regulated. As expected, the treatment of baicalein (16 μM) reduced the protein level of c-Jun, c-Fos, MMP-2 and MMP-9 apparently. In addition, the endothelial cell invasion assay (Figure 6C) and tube formation assay (Figure 6D) were used to evaluate the effect of baicalein on THP-1 CM-induced angiogenesis *in vitro* when AP-1 was overexpressed. We observed that overexpression of c-Jun or c-Fos increased the invasion and tube formation of HUVECs, while treatment with baicalein significantly weakened the angiogenic ability of HUVECs. On the basis of these findings, we proposed that baicalein exerted an anti-angiogenesis effect by inhibiting AP-1 signaling.

### Baicalein inhibited THP-1 CM-induced angiogenesis *in vivo*

**Figure 7:**
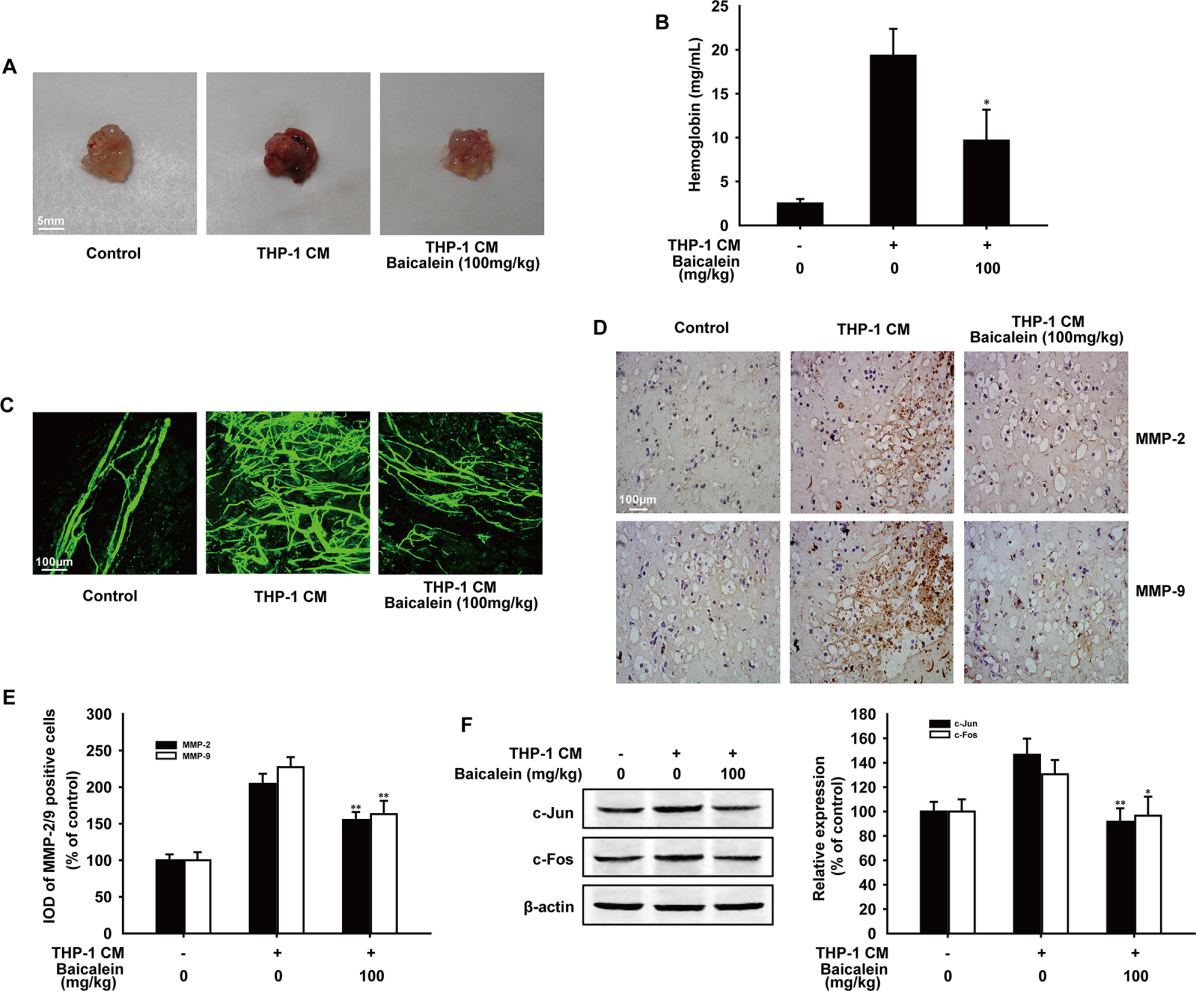
Effect of baicalein on THP-1 CM induced angiogenesis *in vivo* Matrigel containing HUVECs and saline injection (control group) or THP-1 CM was injected subcutaneously to assess angiogenesis *in vivo*. (**A**) Macroscopic appearance of matrigel plugs isolated from each group of mice. (**B**) The hemoglobin content in gel was determined. (*n* = five mice per group) (**C**) The whole-mount of CD31 staining was viewed by laser scanning confocal microscope. (**D**) Representative images of immunohistochemical staining for MMP-2 and MMP-9 of matrigel plugs (100×) (*n* = five mice per group). (**E**) Image pro plus software was used to quantify the IHC images and 10 fields were counted for each mouse. IOD of MMP-2 and MMP-9 positive cells were shown. (**F**) The expression of c-Jun and c-Fos in matrigel plugs were detected by Western blot analysis. Each experiment was performed at least three times. Data are presented as mean ± SD. The comparisons were made relative to THP-1 CM-treated group and significance of difference is indicated as *P < 0.05, **P < 0.01.

To further investigate whether baicalein directly inhibits THP-1 CM-induced angiogenesis *in vivo*, matrigel plug assay was performed. As shown in Figure 7A, matrigel plugs in THP-1 CM-induced group appeared dark-red color, in contrast, plugs in control or baicalein (100 mg/kg) treated group were pale in their color which indicated the formation of blood vessel was suppressed by baicalein. In addition, matrigel plugs from mice in THP-1 CM-induced group had a higher hemoglobin concentration than the control group, while the hemoglobin concentration was reduced by 50.1% with the treatment of baicalein (100 mg/kg) (Figure 7B). Most noticeably, the whole-mount of CD31 staining (Figure 7C) showed that baicalein (100 mg/kg) reduced the vascular density in matrigel plugs obviously compared with THP-1 CM-stimulated group. Furthermore, results of immunohistochemical staining revealed that the protein expression of MMP-2 and MMP-9 in matrigel plugs was also reduced by baicalein. Compared with the THP-1 CM-induced group, the inhibition rate was 49.3% and 64.3%, respectively (Figure 7D). Besides, the expression of c-Jun and c-Fos in plugs was also decreased by baicalein (100 mg/kg) and the inhibition percentage was 55.0% and 33.9%, respectively. Taken together, baicalein inhibited THP-1 CM-induced angiogenesis *in vivo*.

## DISCUSSION

Angiogenesis is promoted by a variety of proangiogenic factors, such as VEGF, basic fibroblast growth factor (bFGF), interleukin-6/8/10 (IL-6/8/10) and granulocyte-macrophage colony-stimulating factor (GM-CSF) [30]. Particularly, VEGF and the activation of the vascular endothelial growth factor receptor (VEGFR) that specifically stimulate ECs proliferation and migration during angiogenesis, have been served as primary targets for anti-angiogenic therapy and approved for clinical use worldwide [31]. However, most VEGF blockers have limitations in wide clinical applications for their undesirable side effects and most patients develop resistance to anti-VEGF/VEGFR therapy, which is associated with inflammation and may result from compensatory signaling pathways activated by inflammatory cytokines [32, 33]. Hence, novel effective anti-angiogenic agents with fewer side effects and wider application are urgent to be developed.

In chronic inflammation, pathological angiogenesis occurs when inflammatory cells, such as the activated or M2 macrophages, secrete MMPs, cathepsins and other cytokines triggering quiescent ECs to proliferate, migrate and form new blood vessels [8, 34]. Here, we used the conditioned media of LPS-stimulated THP-1 cells (THP-1 CM), which contained a variety of inflammation cytokines existing in the inflammation microenvironment, in several *in vitro* and *in vivo* experiments to evaluate the effect of baicalein on angiogenesis. Tube formation represents the beginning of angiogenesis and the rat aortic ring assay can mimic several stages in angiogenesis, including endothelial cell proliferation, migration and microvessel outgrowth, which were used as *in vitro* models to evaluate the effect of baicalein on angiogenesis. As the new blood vessel formation in CAM is similar to angiogenesis, CAM assay was performed as a unique *ex vivo* model to further investigate the anti-angiogenic effect of baicalein. These results revealed that baicalein could suppress THP-1 CM-induced angiogenesis, which was further confirmed by the Matrigel plug assay in nude mice.

ECs proliferation and migration are two crucial steps in angiogenesis. The result of MTT assay ruled out the possibility that baicalein inhibited THP-1 CM-induced angiogenesis was due to the decreased number and vitality of HUVECs. The change of actin cytoskeleton and cytoplasmic stress fibers formation is required for the increased motility of ECs. Morphology observation and actin cytoskeleton staining, along with the results of wound healing and migration assay showed that baicalein inhibited the motility and migration of HUVECs. In addition, the ECM and blood vessel basement membrane degradation is especially needed for ECs migration, which is accelerated by the MMPs [19]. Therefore, we studied the effect of baicalein on MMP-2 and MMP-9, two important members of MMPs. According to gelatin zymography, RT-PCR and Western blot analysis, baicalein inhibited the activity and expression of MMP-2 and MMP-9 in a concentration-dependent manner. All the results implied that baicalein inhibited THP-1 CM-induced angiogenesis by reducing the activity and expression of MMP2/9 to suppress ECs migration.

In inflammation microenvironment, a series of transcription factors can be activated by inflammatory stimulus to increase the expression of pro-angiogenesis factors, such as VEGF, IL-6 and MMPs [35, 36]. The previous study showed that NF-κB and STATs were involved in the inhibition effect of baicalein on inflammation [37, 38]. Therefore, we hypothesized that the inhibition of the main transcription factors activated in inflammation microenvironment may account for the underlying molecular mechanism of baicalein that inhibited the expression of MMP2/9 in THP-1 CM-induced HUVECs. First of all, we used molecular modeling and docking studies to explore whether baicalein can interact with NF-κB, STAT-3 or AP-1 directly. The stable hydrogen bond only existed between baicalein with AP-1 at the Lys5 site and the aromatic ring branched chain of baicalein stretched into the hydrophobic pocket consisted of Lys5, Arg6, Met7, Arg8 and Asn9. On the contrary, molecular distance between the energy minimum molecular pose of baicalein and NF-κB was close enough to form a repulsive force and baicalein was almost distant free from the tertiary structure of STAT-3. Therefore, we focused our attention on AP-1 and to study the possible effect of baicalein. Baicalein inhibited the protein expression of c-Jun and c-Fos, while the mRNA level of them was not influenced by baicalein. These results indicated that baicalein suppressed AP-1 at post-transcription level, which was then confirmed by western blot analysis after HUVECs were treated with CHX for inhibiting de novo protein synthesis and MG132 working as a proteasome inhibitor to inhibit the AP-1 degradation via ubiquitination [39]. The reduction effect of baicalein on c-Jun and c-Fos was abrogated by MG132, which indicated that baicalein could reduce the expression of AP-1 by promoting its proteasomal degradation. In addition, as the nuclear translocation and heterodimerization of c-Jun and c-Fos are two necessary steps for AP-1 to initiate downstream target genes transcription [15], we further studied the inhibition effect of baicalein on AP-1 by immunofluorescence and immunoprecipitation assay. As a result, the reduced nuclear translocation, combination and DNA binding affinity of c-Jun and c-Fos were observed and followed with their expression inhibition in HUVECs treated with baicalein.

In addition, nearly 300 literatures have demonstrated that AP-1 plays a significant role in angiogenesis [40, 41]. To further confirm the important role of AP-1 in the inhibition effect of baicalein on THP-1 CM-induced angiogenesis, c-Jun or c-Fos expression plasmids were used. The overexpression of c-Jun or c-Fos increased MMP-2/9 expression, the migration and tube formation of HUVECs, which were weakened by the treatment of baicalein significantly. Therefore, we proposed that baicalein might exert anti-angiogenesis effect in the inflammation microenvironment by inhibiting the expression and DNA binding affinity of AP-1 *in vitro*. Consequently, whether baicalein can inhibit tumor angiogenesis by modulating AP-1 needs further investigation. Also, further studies will be performed to determine the underlying molecular mechanism of the anti-angiogenic effect of baicalein *in vivo*. What's more, as one of the major active flavanoids found in *Scutellaria baicalensis* Georgi, baicalein has not been used clinically in any therapies or treatments and all its activities were reported in preclinical studies. However, the traditional Chinese medicine *Scutellaria baicalensis* Georgi has been widely used in clinical for many years to treat ischemia, joint discomfort, cancer and various inflammatory diseases [42, 43]. Therefore, baicalein may have a great application potential in clinic and more studies providing further insight into the molecular mechanisms underlying the clinical use of baicalein are urgent in need.

In recent years, to develop therapeutic modalities for preventing angiogenesis in some illnesses such as different types of cancers, ophthalmic sicknesses and inflammation-related diseases, VEGF/VEGFR2 signaling is perhaps the most extensively studied and successfully targeted [31]. Unfortunately, treatment of the VEGF-specific antibody (bevacizumab), does not increase the survival rate of some cancer patients and it is associated with enhanced tumor invasion and metastasis. Therefore, it is preferably that suppressing other compensatory signaling pathways can reduce angiogenesis and be conducive to diseases treatment. The previous study has proved that baicalein could suppress HUVECs proliferation to decrease VEGF-induced angiogenesis by inhibit the activation of VEGFR2 directly [22]. Further, we gave the evidence that baicalein inhibited inflammation-induced angiogenesis by promoting AP-1 degradation then reducing its expression, which may make us know more comprehensively about the anti-angiogenic mechanism of baicalein. Besides, the previous reports were focused on the inhibition effect of baicalein on EC proliferation, while we studied mainly about baicalein inhibited the activation of ECs. As the activation of ECs plays an important role in helping vessel normalization and function, whether baicalein can promote vessel normalization transiently as bevacizumab dose and except MMP2/9, if baicalein can increase endothelial junction proteins, such as VE-cadherin, ZO-1, occluding and claudin-5 needs further exploration. Although, there were many reports demonstrated the anti-inflammation effect of baicalein and baicalein was identified as a lipoxygenase inhibitor by some researchers [24]. As one active component extracted from traditional Chinese medicine, baicalein may exhibit its pharmacological effect through affecting multiple steps in the development of disease and the related mechanism won't be the same in different cell lines and experimental models.

In conclusion, we demonstrated that baicalein could inhibit angiogenesis in inflammatory microenvironment via suppressing the expression of AP-1 for the first time. These findings provided the evidence that baicalein could be developed as a potential angiogenesis inhibitor for the treatment of inflammatory related diseases and had advantages for potential clinical applications in the future.

## MATERIALS AND METHODS

### Reagents and antibodies

Baicalein (C_15_H_10_O_5_, MW: 270.24), isolated from *Radix Scutellariae* Georgi, with the purity of 97% as determined by HPLC, was dissolved at a concentration of 0.1 M in 100% dimethylsulfoxide (DMSO) as a stock solution and stored at −20°C. The working solution was freshly diluted with the basal medium to the final concentration for *in vitro* study and the control group was treated with the same amount of DMSO as used in the corresponding experiments. The final DMSO concentration did not exceed 0.1% throughout the study. *In vivo* study, Baicalein was prepared as intragastric administration (0.5% sodium carboxyl methyl cellulose) by Dr. Xue Ke from College of Pharmacy, China Pharmaceutical University.

DMSO, LPS (*E.coli*: Serotype 055:B5), paraformaldehyde, Triton X-100, Tris, NaCl, EDTA, NP-40, PMSF, NaF, SDS, DTT and fluorescein-5-isothiocyanate (FITC)-conjugated phalloidin were purchased from Sigma Chemical Co. (St. Louis, MO). Sodium carboxyl methyl cellulose (CMC) was obtained from Sinopharm Group Co. Ltd. (Shanghai, China). Dye 4, 6-diamidino-2-phenylindole (DAPI) was purchased from Invitrogen (Carlsbad, CA, USA). Triton X-100 was purchased from Chao Rui Biotech. Co. Ltd. (Shanghai, China). BSA was purchased from Roche Diagnosis Ltd. (Shanghai, China).

Primary antibodies for MMP-2, MMP-9, c-Fos and β-actin were purchased from Santa Cruz Biotechnology (Santa Cruz, CA). Primary antibodies for c-Jun, and LaminA were obtained from Bioworld (St. Louis Park, MN). Primary antibody for β-Tublin was obtained from Cell Signaling Technology (Danvers, MA). Primary antibody for CD31 was obtained from BD Biosciences (Becton Dickinson, Bedford, MA). IRDye^TM^ 800 Conjugated anti-mouse/rabbit second antibody was purchased from Rockland Inc. (Philadelphia, PA, USA).

### Cell culture

HUVECs were isolated from human umbilical cord veins by collagenase treatment as described previously [44]. The harvested cells were grown in medium 199 (Gibco, Grand Island, NY) containing endothelial cell growth supplement (ECGS, 30 μg/ml; Sigma, St. Louis, MO), epidermal growth factor (EGF, 10 ng/ml; Sigma, St. Louis, MO), 20% fetal bovine serum (FBS, Gibco, Grand Island, NY), 100 U/ml penicillin and 100 U/ml streptomycin, pH 7.4. After 3–5 passages, HUVECs were collected for use in all experiments. Human acute monocytic leukemia THP-1 cells were obtained from CBCAS (Cell Bank of the Chinese Academic of Sciences, Shanghai, China) and were cultured in RPMI-1640 medium (Gibco, Carlsbad, CA), supplemented with 10% fetal bovine serum (Gibco, CA, USA), 100 U/ml benzyl penicillin and 100 U/ml streptomycin. All cells were incubated in a humidified atmosphere of 95% air + 5% CO_2_ at 37°C.

### THP-1 conditioned media collection

The conditioned media of LPS-stimulated THP-1 cells (THP-1 CM) containing a variety of inflammation cytokines could be used to simulate the *in vivo* inflammation microenvironment, which was collected as described below and added in each experiment to evaluate the effect of baicalein on inflammation-induced angiogenesis. THP-1 cells were activated with 1μg/ml LPS for 12 h and collected by centrifuging as 500 rpm/min for 5 min, then washed with PBS (PH 7.4) to remove the LPS. Resuspend these LPS-stimulated THP-1 cells with 1% FBS RPMI-1640 medium to 1 × 10^6^ cells/ml. THP-1 CM was collect by centrifuging at 4000 rpm/min for 5 min after the cells were cultured for another 24 h. The resulting THP-1 CM was stored in aliquots at −80°C until use. HUVECs were exposed to regular medium alone or regular medium containing 50% THP-1 CM (regular medium: THP-1 CM = 1:1) with baicalein at indicated concentrations for 24 h before being used in each experiment.

### Tube formation assay

An *in vitro* capillary tube formation assay was performed as described earlier [45], which was used to evaluate the effect of baicalein on the tube formation ability of HUVECs. Briefly, matrigel (Becton Dickinson, Bedford, MA) was thawed and mixed with an equal volume of non-FBS medium. The mixture was transferred into a 96-well plate and rendered to solidify and polymerize at 37°C for 45 min. After pretreated with regular medium (control group) or regular medium containing 50% THP-1CM with various concentrations of baicalein (0, 1, 4 and 16 μM) for 24 h, HUVECs were harvested with trypsin, suspended in 1% FBS medium and seeded onto matrigel. Following 8 h of incubation in 5% CO_2_ at 37°C, the plate was examined for capillary tube formation under an inverted microscope. Tubular structures were photographed and quantified by manual counting of tube numbers. Five randomly chosen fields were analyzed for each well.

### Rat aortic ring assay

Rat aortic ring assay was performed as described previously to test the effect of baicalein on the formation of microvessel outgrowth from rat aorta explants [46]. The thoracic aorta was harvested from male Sprague-Dawley rats (six weeks old) and cut into 1 mm slices and set in a 24-well plate. Prepare the clotting media containing M199^+^ (M199 with 200 U/ml penicillin and 200 μg/ml streptomycin), 0.3% fibrinogen and 0.5% ε-amino-η-caproic acid (ACA; Sigma, St. Louis, MO). Then the growth media consisting of M199^+^ with 20% FBS and 0.5% ACA was added to each well. One to two days later, cells started to sprout from the explants, forming microvessel-like structures. After three days of growth, six rings were used as a group and fed with 1 ml of M199^+^ alone or M199^+^ containing 50% THP-1 CM with various concentrations of baicalein (0, 6.25, 12.5 and 25 μM) as indicated. Plates were then stored in the incubator at 37°C. After seven days, the sprouting microvessels in five randomly chosen fields were measured and photographed under a microscope for each group.

### Chicken chorioallantoic membrane (CAM) assay

CAM assay used as a unique *ex vivo* model to investigate the effect of baicalein on the process of new blood vessel formation was performed according to the method described with modification [47]. Briefly, fertilized chicken eggs were incubated at 37°C for 9 days. After this incubation, a small hole was punched on the broad side of the egg, and a window was carefully created through the egg shell. THP-1 cells (1 × 10^6^ cells/embryo) pretreated with LPS for 12 h, were placed on the exposed CAM. Sterilized filter paper disks (5 mm × 5 mm) saturated with baicalein (0, 4, 16 and 64 ng/CAM), were placed on the CAMs. They were then incubated at 37°C for another 2 days. Then an appropriate volume of 10% fat emulsion (Intralipose, 10%) was injected into the embryo chorioallantois for observing the density and length of vessels toward the CAM face. Neovascular zones under the filter paper disks were observed and photographed by a digital camera at × 5 magnification. The number of newly growth vessels was counted on digitalized pictures.

### Cell viability assay

The MTT assay was used to evaluate the viability of endothelial cells affected by baicalein. HUVECs were plated at a density of 2 × 10^5^ cells per well into 96-well plates in medium with 10% FBS. After overnight growth, HUVECs were incubated in regular medium or regular medium containing 50% THP-1CM with different concentrations of baicalein (0, 1, 4 and 16 μM) for 24 h in 5% CO_2_ incubator at 37°C. Then 20 μl of 0.5% MTT were added to the medium and incubated for 4 h. The supernatant was removed and 100 μl DMSO were added to dissolve the precipitate. Absorbance was measured at 570 nm.

### Immunofluorescence staining of the actin cytoskeleton

The actin cytoskeleton staining was performed to test the motility of endothelial cells. HUVECs grown on coverslips were incubated in regular medium or regular medium containing 50% THP-1CM with different concentrations of baicalein (0, 1, 4 and 16 μM) as indicated for 24 h. Cells were fixed with 4% paraformaldehyde for 20 min and permeabilized for 10 min in 0.2% Triton X-100, then incubated with 3% BSA in PBS to block nonspecific binding. Cells were subsequently processed by incubation with FITC-conjugated phalloidin (specific for F-actin staining), 1:30 dilution for 1 h at 37°C. Then, cells were washed three times with PBS and stained with DAPI. Finally, the slips were mounted with anti-fade reagent (Molecular Probes, Inc., Eugene, OR) and photographed with a confocal laser scanning microscope (Fluoview FV 1000, Olympus, Tokyo, Japan).

### Wound healing assay

Wound healing assay was used to investigate the effect of baicalein on cell migration ability. HUVECs were seeded into six-well plates to reach a confluence, which was wounded with a yellow pipette tip. After cells were rinsed with PBS (pH 7.4), 1% FBS medium (control group) and 1% FBS medium containing 50% THP-1CM with baicalein (0, 1, 4 and 16 μM) as indicated were added into the wells. The plates were incubated as above and photographed at 0, 12, and 24 h. The migrated distance of cells was measured and five randomly chosen fields were analyzed for each well.

### Endothelial cell invasion assay

The transwell assay was used to study the cell invasion ability affected by baicalein. The invasion ability of HUVECs was assayed using a transwell chamber (6.5 mM in diameter, 8 mM pore-size, CorningCostar, Cambridge, MA). Firstly, transwell chambers were loaded with 0.1 ml matrigel (Becton Dickinson, Bedford, MA) at 37°C for 1 h. HUVECs were incubated in regular medium or regular medium containing 50% THP-1CM with different concentrations of baicalein (0, 1, 4 and 16 μM) as indicated for 24 h. Cells were then trpsinized and suspended at a final concentration of 5 × 10^5^ cells/ml in serum-free M199. Cell suspension was loaded into each of the upper wells and regular medium or regular medium containing 50% THP-1CM was added into in the lower compartment. Following incubation at 37°C in 5% CO_2_ for 24 h, the non-migratory cells on the upper surface were removed by a cotton swab. The invasive cells on the lower surface were fixed with 100% methanol and stained with hematoxylin and eosin. The migrated cells were quantified by manual counting and five randomly chosen fields were analyzed for each group.

### Gelatin zymography

Gelatin zymography was performed using MMP Zymography Assay Kit to investigate the gelatinolytic activity of MMP-2 and MMP-9 secreted by HUVECs (SIGMA AMRESCO TAO, Shanghai, China). HUVECs were pretreated with THP-1CM and baicalein for 24 h as above, and the supernatants were collected to prepare samples with loading buffer. Proteins were subjected to 10% SDS-PAGE containing 0.1% gelatin. After electrophoresis, the gels were washed twice with rinsing buffer containing 50 mM Tris-HCl (pH 7.6), 5 mM CaCl_2_, 1 μM ZnCl_2_, and 2.5% Triton X-100 to remove the SDS. Then gels were incubated for 48 h in 50 mM Tris-HCl buffer containing 5 mM CaCl_2_, 1 μM ZnCl_2_. After these, the gels were stained with 0.1% Coomassie Brilliant Blue R-250 for 1 h and destained with 10% acetic acid and 10% methanol. Enzyme-digested regions were appeared as white bands and the gels were photographed using the GeneGenius Image and Analysis System (Syngene, Cambridge, UK).

### Molecular modeling and docking studies

Molecular modeling and docking studies were used to explore the direct interaction of baicalein with transcription factors. The crystal structure of NF-κB (pdb code: 2RAM), STAT-3 (pdb code: 1BG1) and AP-1 (pdb code: 2H7H) were prepared by the Protonate 3D tool in MOE2009 and all the water molecules were removed. Hydrogen atoms were added using MOE. The structure of baicalein was modeled and minimized in MOE. Docking simulations were carried out in the CDOCKER module implemented in Discovery Studio 2.5.

### Preparation of whole cell lysates and cytosolic and nuclear extracts

The whole cell lysates was isolated by Pierce RIPA buffer added with protease inhibitors (1 mM Phenylmethanesulfonyl fluoride, 0.1 mM dithiothreitol, 0.1 Mm NaF, 0.1 mM Leupeptin), incubated on ice for 55 min to allow cells to swell and then centrifuged at 12,000 rpm for 30 min at 4°C. The supernatants were saved as the whole cell lysates and measured using the BCA protein assay method with Varioskan spectrofluorometer and spectrophotometer (Thermo) at 562 nm.

Cytosolic and nuclear protein extracts were prepared according to the modified method as described below. After washing with PBS, HUVECs were trypsinized and harvested in tubes by scraping in ice-cold PBS and collected by centrifugation at 2,500 rpm for 5 min at 4°C. Cells were lysed with buffer A (10 mM Hepes-KOH (pH 7.9), 10 mM KCl, 0.1 mM EDTA, 0.5% Nonidet P-40, 1 mM dithiothreitol, 0.5 mM phenylmethylsulfonyl fluoride), incubated on ice for 13 min to allow cells to swell and then centrifuged at 12,000 rpm for 15 min at 4°C. The supernatants were saved as the cytoplasmic fractions. The nuclear pellets were washed three times with buffer A, then resuspended with high-salt buffer (20 Mm Hepes, 0.5 M KCl, 1 mM EDTA, 1 mM dithiothreitol, 1 mM phenylmethylsulfonyl fluoride, pH 7.9) for 45 min on ice and then centrifuged at 12,000 rpm for 15 min at 4°C. One part of the cytosolic and nuclear fractions was subjected to immunoblot analysis. The rest of the nuclear extract was used for electrophoretic mobility-shift assay (EMSA).

### Western blot analysis

Western blot analysis was used to detect the expression of target protein. Protein samples were separated by 10% SDS-PAGE and transferred onto nitrocellulose membranes. The membrane was blocked with 1% BSA in PBS at 37°C for 1.5 h and incubated with indicated antibodies overnight at 4°C, followed by IRDyeTM800 conjugated secondary antibody for 1 h at 37°C. Detection was performed by the Odyssey Infrared Imaging System (LI-COR Inc., Lincoln, Nebraska).

### Quantitative real-time PCR analysis

Quantitative real-time PCR analysis was used to detect the RNA level in HHUVECs. Total RNA was isolated with Tripure Isolation Reagent (Roche, Mannheim, Germany). The real-time PCR (RT-PCR) kit was purchased from TaKaRa Biotechnology Co. Ltd. (Dalian, China) and the FastStart Universal SYBR Green Master (Rox) was purchased from Roche (Mannheim, Germany). Reactions were conducted with 1 μl of RT-PCR reaction cDNA, 0.5 μl each forward and reverse primers (10 μM), 8 μl distilled/deionized ddH_2_O, and 10 μl SYBR Green Master (Rox). Samples were run on the ABI 7500 Real-Time PCR system as follows: 2 min at 50°C, then 10 min at 95°C, followed by 45 cycles of 95°C for 15 s, and 60°C for 1 min. Each reaction was done in triplicate, and the threshold values (Cs) for each mRNA were subtracted from that of β-actin mRNA and averaged and converted from log-linear to linear terms. Data were analyzed with the SDS 2.1 program. The primers in the reaction were used as follows: MMP-2 (forward: 5′-CAGGCTCTTCTCCTTTCACAAC-3′, reverse: 5′-AA GCCACGGCTTGGTTTTCCTC-3′); MMP-9 (forward: 5′-GCAGAGGAATACCTGTACCGC-3′, reverse: 5′-AGG TTTGGAATCTGCCCAGGT-3′); c-Jun (forward: 5′-ATC AAGGCGGAGAGG AAG CG-3′, reverse: 5′-TGAGCA TGTTGGCCGTGGAC-3′); c-Fos (forward: 5′-CTGGCGT TGTGAAGACCA T-3′, reverse: 5′-TCCCTTCGGATTCT CCTTTT-3′); β-actin (forward: 5′-CTGTCCCTGTATGCC TCTG-3′, reverse: 5′-ATGTCA CGCACGATTTCC-3′).

### Immunofluorescence microscopy

Immunofluorescence assay was performed to investigate the effect of baicalein on protein expression and translocation. HUVECs were grown on coverslips and pretreated with regular medium alone or 50% THP-1CM with different concentrations of baicalein (0, 1, 4 and 16 μM) as indicated for 24 h. Cells were fixed with 4% paraformaldehyde for 20 min, permeabilized in 0.2% Triton X-100 for 10 min, and incubated with 3% BSA in PBS for 1 h to block nonspecific binding. After incubated with primary antibodies (c-Jun and c-Fos) overnight at 4°C, cells were exposed to anti-rabbit FITC conjugated or anti-mouse tetramethyl rhodamine isothiocyanate conjugated (TRITC) secondary antibodies (1:1000, Invitrogen, Carlsbad, CA, USA). Then, cells were washed three times with PBS, stained with DAPI and the slips were mounted with anti-fade reagent (Molecular Probes, Inc., Eugene, OR). Finally, cells were observed and photographed with a confocal laser scanning microscope (Fluoview FV 1000, Olympus, Tokyo, Japan).

### Immunoprecipitation

Supernatants of HUVECs lysates were incubated with c-Fos antibody for 1 h at 4°C, and then added 20 μl of protein G/A agarose beads (Santa Cruz Biotechnology, St. Louis Park, Minnesota, US) overnight at 4°C. Beads were washed four times with cell lysis buffer and bound proteins were eluted with 2 × loading sample buffer and analyzed by Western blot with c-Jun antibody.

### Electrophoretic mobility shift assays (EMSA)

EMSA was performed to study the DNA binding affinity of AP-1 in HUVECs. Nuclear extracts preparation was conducted as described above. EMSA was performed with a non-radioactive (biotin label) gel shift assay according to the manufacturer's protocol (Beyotime Institute of Biotechnology, Haimen, China). The AP-1 consensus oligonucleotides probes labeled with biotin (5′-CGCTTGATGACTCAGCCGGAA-3′ and 3′-GCGAACTACTGAGTCGGCCTT-5′) were annealed to their complementary oligonucleotides and incubated with nucleoproteins for 40 min at 25°C. Samples were run on a 6% polyacrylamide gel, which was transferred into Nylon member and then blocked and washed. Bands were detected by chemiluminescent method.

### Transient transfection

Transient transfection was performed to make c-Jun or c-Fos over-expressed in HUVECs then to further investigate the effect of baicalein on AP-1. HUVECs were seeded at a density of 1.5 × 10^5^ cells per well into 6-well plate in medium with 10% FBS. The transient transfection assay was performed by using PolyJet™ DNA *in Vitro* Transfection Reagent (SignaGen, MD) according to the manufacturer's protocol. Firstly, 3 μl Transfection Reagent was diluted in 50 μl M199 medium gently, and 1 μg c-Jun or c-Fos plasmid was mixed in 50 μl M199 medium gently. Then, the diluted oligomer was combined with the diluted Transfection Reagent gently and incubated for 20 min at room temperature. The oligomer-Transfection Reagent complexes were added to each well and mixed gently by rocking the plate back and forth. The plate was then incubated at 37°C in a CO_2_ incubator and the medium was changed after 12 h.

### *In vivo* angiogenesis

The matrigel plug assay was performed to assess *in vivo* angiogenesis as described previously [48]. Briefly, 3 to 4-wk-old BALB/c-nude mice (Slaccas Shanghai Laboratory Animal Co., Ltd., Shanghai, China) were maintained in a pathogen-free environment (23 ± 2°C, 55 ± 5% humidity) on a 12 h light/12 h dark cycle with food and water supplied adlibitum throughout the experimental period. Mice were injected subcutaneously on the flank with 500 μl matrigel (BD Biosciences) containing HUVECs (2 × 10^6^ cells per gel), heparin (10 U per gel) and 100 μl saline injection (control group) or 100 μl THP-1 CM. For mice in the THP-1 CM-induced group and baicalein treated group, THP-1 CM (200 μl per injection) were subcutaneously administered daily for 10 days. Mice were killed and the gel plugs were excised, photographed, viewed whole-mount of CD31 staining, and then analyzed by immunohistochemistry of MMP-2 or MMP-9 staining, as well as ground and detected by western blot assay. Alternatively, plugs were homogenized in 1 ml PBS buffer, centrifuged and the content of hemoglobin in the supernatant was by Drabkin's reagent (Sigma-Aldrich). Animal study and euthanasia were carried out in strict accordance with the recommendations in the Guide for the Care and Use of Laboratory Animals of the National Institutes of Health. The protocol was approved by the Committee on the Ethics of Animal Experiments of the China Pharmaceutical University.

### Statistical analysis

The data shown in the study were obtained in at least three independent experiments and all data in different experimental groups were expressed as the mean ± SD. Statistical analyses were performed using a One-Way ANOVA, with post-Hoc analysis. Details of each statistical analysis used are provided in the figure legends. Differences with *P* values < 0.05 were considered statistically significant.

## Abbreviations

HUVECs: human umbilical vein endothelial cells
ECs: endothelial cells
VEGF: vascular endothelial growth factor
MMPs: matrix metalloproteinases
ECM: extracellular matrix
AP-1: activator protein-1
MAPK: mitogen-activated protein kinases
BSA: bovine serum albumin
DAPI: diamidino-phenyl-indole
DMSO: dimethylsulfoxide
LPS: lipopolysaccharides
PBS: phosphatebuffered saline
CAM: Chicken chorioallantoic membrane
CHX: cycloheximide
